# Measuring Belief in Conspiracy Theories: The Generic Conspiracist Beliefs Scale

**DOI:** 10.3389/fpsyg.2013.00279

**Published:** 2013-05-21

**Authors:** Robert Brotherton, Christopher C. French, Alan D. Pickering

**Affiliations:** ^1^Department of Psychology, Goldsmiths University of LondonLondon, UK

**Keywords:** conspiracy theories, conspiracist ideation, individual differences, personality, psychometric instrument, scale design

## Abstract

The psychology of conspiracy theory beliefs is not yet well understood, although research indicates that there are stable individual differences in conspiracist ideation – individuals’ general tendency to engage with conspiracy theories. Researchers have created several short self-report measures of conspiracist ideation. These measures largely consist of items referring to an assortment of prominent conspiracy theories regarding specific real-world events. However, these instruments have not been psychometrically validated, and this assessment approach suffers from practical and theoretical limitations. Therefore, we present the Generic Conspiracist Beliefs (GCB) scale: a novel measure of individual differences in generic conspiracist ideation. The scale was developed and validated across four studies. In Study 1, exploratory factor analysis of a novel 75-item measure of non-event-based conspiracist beliefs identified five conspiracist facets. The 15-item GCB scale was developed to sample from each of these themes. Studies 2, 3, and 4 examined the structure and validity of the GCB, demonstrating internal reliability, content, criterion-related, convergent and discriminant validity, and good test-retest reliability. In sum, this research indicates that the GCB is a psychometrically sound and practically useful measure of conspiracist ideation, and the findings add to our theoretical understanding of conspiracist ideation as a monological belief system unpinned by a relatively small number of generic assumptions about the typicality of conspiratorial activity in the world.

## Introduction

A conspiracist belief can be described as “the unnecessary assumption of conspiracy when other explanations are more probable” (Aaronovitch, 2009, p. 5). Substantial numbers of people endorse conspiracy theories proposing that the U.S. government orchestrated the 9/11 terrorist attacks (Stempel et al., 2007; Swami et al., 2010), that the British government is covering up its own role in the 7/7 bombings (Soni, 2007), and that the assassination of President John F. Kennedy was not the work of Lee Harvey Oswald alone (Goertzel, 1994; McHoskey, 1995), to name but a few popular conspiracy theories (see McConnachie and Tudge, 2008). Such beliefs are usually unsubstantiated and implausible, but are often regarded as harmless (e.g., Clarke, 2002). Yet some conspiracy theories are associated with negative outcomes; conspiracist beliefs about the origin and treatment of HIV/AIDS have been found to detrimentally affect attitudes toward preventative measures and adherence to treatment programs (Bogart et al., 2010), and conspiracist fears concerning the safety of childhood vaccinations have played a role in declining vaccination rates (Salmon et al., 2005; Kata, 2010; Offit, 2011). Other conspiracy theories can lead to social and political disengagement (Butler et al., 1995; Jolley and Douglas, 2013) and may help to foster political extremism (Bartlett and Miller, 2010).

To date only a small handful of studies have begun to investigate the formation and maintenance of conspiracy beliefs, largely examining the role of individual differences in broad personality traits and cognitive styles (e.g., Goertzel, 1994; McHoskey, 1995; Abalakina-Paap et al., 1999; Leman and Cinnirella, 2007; Douglas and Sutton, 2008, 2011; Swami et al., 2010, 2011, 2013; Darwin et al., 2011; Swami, 2012). The handful of available findings suggests that conspiracist beliefs are linked with traits such as low interpersonal trust, paranoia, and receptivity to other unusual beliefs. The relationships between conspiracy beliefs and more established psychological measures or general models of personality are largely unknown. Some research has examined correlations between conspiracist ideation and the Big-5. Weak but significant relationships have been reported with higher openness and lower agreeableness, however some studies have failed to replicate these relationships (see Swami et al., 2010, 2011, 2013; Swami and Furnham, 2012).

One robust finding to emerge from the limited existing literature is that individuals who endorse one conspiracy theory tend to endorse others, including unrelated theories (Goertzel, 1994; Swami et al., 2010, 2011, 2013), fictitious theories made up by psychological researchers (Swami et al., 2011), and even mutually contradictory theories (Wood et al., 2012). In total, these findings suggest that endorsement of conspiracy theories is not exclusively a result of rational evaluation of the evidence relating to each specific conspiracist claim; rather it appears there are stable individual differences in the general tendency to engage with conspiracist explanations for events. This trait has been termed “conspiracist ideation” (Swami et al., 2011).

Much more research is required to illuminate the psychology of conspiracist ideation, and the number of recent publications on the topic (e.g., Leman and Cinnirella, 2007; Douglas and Sutton, 2008, 2011; Bogart et al., 2010; Swami et al., 2010, 2011, 2013; Darwin et al., 2011; Newheiser et al., 2011; Swami, 2012; Wood et al., 2012; Jolley and Douglas, 2013) suggests that research is gaining momentum. However, research is limited by the lack of a validated measure of individual differences in conspiracist ideation. To produce a coherent body of research it is necessary to devise a valid and psychometrically sound measure which can be used across a variety of empirical contexts.

### Previous approaches toward measuring conspiracist beliefs

Several scales have been created in an attempt to measure individual differences in conspiracist ideation. The dominant approach has been to devise a short self-report questionnaire assessing belief in a small number of conspiracy theories concerning real-world events and situations. Measures have consisted of between six and thirty items with each item referring to a specific currently popular claim of conspiracy. Common subjects include the assassination of President John F. Kennedy, the spread of HIV/AIDS, and the moon landing (Goertzel, 1994; Abalakina-Paap et al., 1999; Leman and Cinnirella, 2007; Darwin et al., 2011; Douglas and Sutton, 2011; Swami et al., 2013). Participants rate their endorsement of each item on a Likert-type rating scale ranging from certain disbelief to certain belief.

There has been little attempt to assess the psychometric properties of any existing measure; most studies have employed novel measures, with little consideration of the psychometric properties of the measure beyond noting the value of Cronbach’s alpha. In addition, no measure has yet been adopted by researchers other than the original authors. This situation presents practical and theoretical problems. Despite the apparent homogeneity of this approach toward measuring conspiracist ideation, idiosyncrasies in item content could influence participant responses. Each scale represents a unique sub-set of currently prominent conspiracy theories; for example, climate-change conspiracy theories feature in some scales (Douglas and Sutton, 2011) but not others (Swami et al., 2010). In addition, items from different scales referring to the same conspiracy theories may not be directly comparable due to differences in wording. Consider two items concerning conspiracy theories alleging the cover-up of extraterrestrials: “*Governments are suppressing evidence of the existence of aliens*” (Douglas and Sutton, 2011; Wood et al., 2012), and “*Area 51 in Nevada, U.S., is a secretive military base that contains hidden alien spacecraft and/or alien bodies*” (Swami et al., 2010). Although both items implicitly refer to the same theory, the differences in tone and specificity may systematically bias responses. It is possible therefore that existing scales do not produce equivalent or directly comparable measures of conspiracist ideation.

This practical problem could be resolved if researchers adopted a single standardized measure. However, any such measure would likely be unsuitable for cross-cultural research as responses may be confounded by the cultural familiarity and relevance (or lack thereof) of the selected theories (Byford, 2011). For example, conspiracy theories concerning the July 7, 2005, bombings in London are relatively well-known within the U.K., but are likely to be less familiar elsewhere. In addition, such a measure would require modification over time as particular conspiracy theories fade from popular awareness and new theories arise in response to world events.

A more fundamental theoretical problem is that existing scales assess attitudes toward a limited number of specific conspiracy theories, yet their intended purpose is to provide a measure of individuals’ generalized tendency toward conspiracy theorizing. A successful measure of this overall conspiracist ideation ought to reflect the entire spectrum of conspiracism. However, any measure referring to specific conspiracy theories faces the problem of selecting a small and arbitrary sub-set of real-world conspiracy theories out of a virtually infinite pool, with the assumption that the selected items are representative of the individual’s overall level of conspiracist ideation. While there is evidence that conspiracist ideation is a unidimensional construct (Goertzel, 1994; Swami et al., 2011; Wood et al., 2012), the content validity of short and potentially unrepresentative measures has thus far gone untested.

### Measuring generic conspiracist beliefs

The issues discussed so far raise concerns over the comparability and generalizability of the handful of existing psychological findings, and the theoretical validity and practical utility of any measure which explicitly refers to prominent real-world conspiracy theories. An alternative and preferable approach toward measuring conspiracist ideation may be to assess generic conspiracist beliefs (GCB). Recent theoretical advances suggest that beliefs in specific conspiracy theories are a product of a smaller set of more GCB. Wood et al. (2012) demonstrated that endorsement of various specific conspiracy theories about the death of Osama Bin Laden is predicted by acceptance of the more general claim that the U.S. government is hiding some important information about the Bin Laden raid. Thus, assessing an individual’s attitude toward a single generic statement can provide a valid and economical indication of beliefs about numerous specific conspiracy theories.

It would be possible to produce a yet more generic scale item concerning the more abstract idea that governments routinely hide information about the deaths of public figures in order to deceive the public. Endorsement of this abstract claim would presumably provide a valid indication of an individual’s beliefs about many popular governmental assassination conspiracy theories, such as those concerning Osama Bin Laden, Princess Diana, and President John F. Kennedy. To take another example, rather than referring explicitly to conspiracy theories of the 9/11 attacks, a non-specific item would have individuals rate their acceptance of the more generic belief that conspiracy within governments to secretly perpetrate terrorist activities on their own citizens is commonplace. In sum, a scale which samples a representative range of these kinds of GCB would assess individuals’ beliefs about the typicality of conspiratorial activity in the real world removed from the context of specific historical events.

Taking this generic, non-event-based approach toward measuring conspiracist ideation can potentially overcome the previously mentioned theoretical and practical problems associated with measures referring to specific popular conspiracy theories. While constructing a measure which refers to specific real-world conspiracy theories necessitates arbitrarily selecting a small sub-set of conspiracy theories and thus compromising content validity, a generic measure could represent the entire spectrum of conspiracist ideation in an economical way by identifying and reflecting the most important generic beliefs which support beliefs in numerous specific conspiracy theories. While a measure referring to currently popular conspiracy theories will require modification as fashions in popular conspiracy theorizing change, a generic measure will remain an appropriate measurement device over time. In addition, by decontextualizing conspiracist beliefs, a generic measure can provide a measurement device suitable for any sample population.

To date, no measure of conspiracist ideation has been designed from the bottom-up, endeavoring to first identify the most important facets of conspiracism which a successful measure should represent, and to provide a measure which represents these facets. Our research was designed to produce such a measure.

### Overview of the present studies

We developed and validated the GCB scale, a novel measure of individual differences in generic conspiracist ideation, across four studies with diverse samples. Informed consent was obtained from all participants and ethical approval for the research was granted by the Goldsmiths, University of London Department of Psychology Ethics Committee.

Study 1 details the first step toward creating a measure of generic conspiracist ideation, which was to identify the most important aspects of conspiracism which should be represented in the measure. To ensure content validity, a measure should sample from the entire gamut of conspiracist themes; however, a short measure is desirable so as to avoid practical problems associated with lengthy questionnaires such as participant frustration, careless responding, drop-out, and reluctance to take part in future studies (see Schmidt et al., 2003; Donnellan et al., 2006). To reconcile the conflicting requirements of brevity and completeness, a lengthy measure consisting of 75 generic conspiracist items was administered to an international sample of the general public and subjected to exploratory factor analysis (*N* = 489) to identify the underlying dimensions of conspiracism which a successful measure should represent. The 15-item GCB scale was created based on the results of this initial study, and the subsequent studies demonstrated the psychometric validity of this measure.

Study 2 aimed to establish content and criterion-related validity, as well as test-retest reliability, using a sample of undergraduate students (*N* = 235) as is typical of most psychological research. To this end, the factor structure of the scale, and its relationships with other measures of conspiracist ideation were examined. Study 3 aimed to establish convergent validity by examining how well the GCB relates to interpersonal trust, anomie, paranormal beliefs, and delusional ideation – individual difference factors previously found to correlate with belief in conspiracy theories (Goertzel, 1994; Darwin et al., 2011; Swami et al., 2011, 2013) – using a sample of the general public (*N* = 208). Study 4 aimed to establish discriminant validity by demonstrating the absence of relationships between the GCB and measures of other theoretically unrelated constructs using a separate sample of the general public (*N* = 194). These constructs consisted of sensation-seeking, emotional intelligence, and the Big Five factors.

## Study 1: Identification of Essential Facets of Conspiracism through Exploratory Factor Analysis

### Method

#### Participants and procedure

To recruit a large and diverse sample not composed exclusively of undergraduate psychology students, volunteers were requested via a blog post on psychologytoday.com and a public “Psychology of the Paranormal” emailing list. In all, 500 participants (225 females, 269 males; no gender data for 6 respondents) completed the questionnaire. Data from 11 participants missing data for 2 or more items were omitted (total valid *N* = 489). Age ranged from 18 to 87 years (*M* = 35.9; SD = 13.9). The majority of participants indicated that they were British (43.8%) or American (26.4%). Other nationalities accounted for 25.6% of the sample (4.2% did not provide nationality information). No reward was offered for taking part. Self-selected respondents completed the questionnaire online via a web-based interface.

#### Measures

##### Conspiracist beliefs scale (long-form)

We designed a novel questionnaire consisting of 75 items intended to reflect generic (i.e., non-event-based) conspiracist beliefs. Items were generated by reviewing the academic and popular literature on conspiracy theories. Each item referred to a generic belief which could support belief in various real-world conspiracy theories. To produce generic items, non-specific descriptors such as “certain organizations,” “the government,” and “significant events” were used in place of references to specific entities or events which are the subject of popular conspiracy theories, such as the U.S. government and the 9/11 attacks. Effort was made to cover a diverse and fully representative range of conspiracist claims. Examples include the possibility of terrorist attacks being covertly directed by a country’s own government, clandestine use of mind-control technology, and concealment by some organization of evidence of extraterrestrial visitation (see Table 1 for exact wording of all items). Participants rated items on a 5-point Likert-type scale, with a qualitative label associated with each point (1: *definitely not true*; 2: *probably not true*; 3: *not sure/cannot decide*; 4: *probably true*; 5: *definitely true*).

**Table 1:** **Conspiracist Beliefs scale (long-form) items and factor loadings**.

	Factor					
	GM	ET	MG	PW	CI	FUF
2. The government has employed people in secret to assassinate others	**0.80**	0.08	−0.10	−0.16	0.01	0.49
48. Government agencies have been secretly involved in the assassination of their own citizens	**0.80**	−0.01	0.02	−0.11	0.04	0.61
61. The deaths of certain high-profile public figures have been the result of covert, government-sanctioned operations	**0.75**	0.07	0.06	0.04	−0.01	0.68
20. Certain world leading political figures who died untimely deaths were in fact “taken out” by government operatives	**0.72**	0.07	0.02	−0.01	−0.02	0.65
55. Some of the people thought to be responsible for acts of terrorism were actually set up by those responsible	**0.65**	−0.04	0.10	0.21	−0.14	0.68
19. For strategic reasons, the government permits certain terrorist activities to occur which could otherwise be prevented	**0.63**	−0.02	−0.05	**0.33**	−0.07	0.71
69. Some acts of terrorism, which have resulted in the deaths of many civilians, have been secretly directed by government operatives	**0.61**	−0.12	0.10	0.31	−0.09	0.71
57. High-level politicians have had certain people killed in order to prevent embarrassing events from becoming publicly known	**0.58**	−0.03	0.01	0.15	0.05	0.66
38. Government agencies secretly keep certain outspoken celebrities and citizens under constant surveillance	**0.56**	0.04	0.03	−0.16	0.19	0.52
34. The government keeps many important secrets from the public	**0.53**	0.08	−0.04	−0.21	0.30	0.52
9. Some individuals thought to be responsible for the assassination of public figures were set up by the group responsible	**0.52**	0.05	0.04	0.18	0.00	0.67
15. The government lies about their knowledge of terrorist activities	**0.49**	−0.08	−0.14	0.09	**0.36**	0.61
22. There are ongoing, hidden efforts to marginalize, control, or destroy certain groups of people through the use of political policies	**0.45**	−0.10	0.12	0.01	**0.33**	0.68
1. Certain celebrities and/or public figures actually faked their own deaths in order to escape the spotlight	**0.41**	0.08	−0.20	0.26	−0.03	0.43
27. Viruses and/or diseases have been deliberately disseminated to infect certain populations	**0.37**	0.02	−0.03	0.24	0.06	0.57
4. The government has staged important societal events in order to manipulate voters	**0.36**	−0.05	0.09	0.10	0.18	0.59
12. The government fakes evidence relating to significant world events to deceive citizens	0.31	−0.01	0.12	0.15	0.28	0.73
30. Evidence of alien contact is being concealed from the public	0.02	**0.91**	−0.05	−0.12	0.14	0.63
53. Evidence of alien presence on earth is being covered up	0.02	**0.90**	−0.04	−0.03	0.07	0.66
25. Secret organizations communicate with extra terrestrials, but keep this fact from the public	0.09	**0.87**	−0.06	0.05	−0.05	0.64
29. Some UFO sightings and rumors are planned or staged in order to distract the public from real alien contact	0.09	**0.76**	−0.03	0.06	0.04	0.68
10. Some existing technologies are the result of reverse engineering alien technology	−0.02	**0.73**	0.02	0.09	0.02	0.63
37. Space missions are deliberately sabotaged so that the public does not learn of existing alien activity in the solar system	0.00	**0.61**	0.03	**0.36**	−0.17	0.66
58. Movies and TV shows featuring aliens are a way of preparing the population for the news that aliens are real and have visited earth	−0.12	**0.58**	0.27	0.15	−0.05	0.64
59. The power held by heads of state is second to that of small unknown groups who really control world politics	0.08	−0.02	**0.88**	−0.04	0.00	0.80
11. A small, secret group of people is actually in control of the world economy	−0.11	0.04	**0.87**	−0.01	0.05	0.75
70. Certain significant world events have been the result of the activity of a small group who secretly manipulate world politics	0.17	−0.03	**0.87**	−0.08	−0.01	0.80
42. A small, secret group of people is responsible for making all major world decisions, such as going to war	−0.10	−0.01	**0.86**	0.08	0.05	0.78
68. Members of a secret group have infiltrated governments and powerful organizations in order to 1 day bring their group to the point of global control	0.04	0.03	**0.76**	0.04	−0.02	0.75
64. Many well-known celebrities, politicians, and wealthy people are members of a secret society which has control over our lives	−0.07	0.08	**0.67**	0.15	−0.03	0.70
14. Large, influential industries are in fact tightly controlled by a small, secret group of people	0.07	−0.02	**0.66**	−0.03	0.17	0.63
44. Small groups of people are in possession of secret knowledge which would change our understanding of the world, and are deliberately keeping it hidden	0.09	0.17	**0.38**	0.05	0.28	0.80
40. Secret organizations have access to large amounts of personal data on every citizen and sell it to the government	0.09	0.17	**0.38**	0.05	0.28	0.66
50. The rapid spread of certain viruses and/or diseases is the result of the deliberate, concealed efforts of some organization	0.10	0.05	0.00	**0.68**	−0.02	0.73
36. Experiments involving new drugs are carried out on the general public without their knowledge or consent	−0.03	0.04	0.09	**0.66**	0.07	0.75
18. Cures for certain deadly and common diseases exist, but are being deliberately withheld	−0.13	0.04	0.09	**0.65**	0.07	0.73
7. Certain natural disasters have in fact been the result of secret testing of powerful and advanced technology with unknown capabilities	0.08	0.14	−0.06	**0.64**	−0.07	0.65
41. The pharmaceutical industry administers harmful treatments without people’s consent in order to keep people sick and boost drug sales	0.02	0.12	0.14	**0.63**	−0.07	0.73
23. Technology with mind-control capacities exists and is currently being used on people without their knowledge	−0.01	0.14	0.09	**0.62**	0.00	0.73
24. A lot of information about diseases and treatments is withheld from the public	−0.06	−0.12	−0.07	**0.61**	**0.48**	0.75
8. The government withholds a lot of information about diseases and their treatments from the public	−0.02	0.02	−0.06	**0.56**	**0.32**	0.72
33. Certain chemicals are put in the water supply in order to control the people	−0.13	0.18	0.26	**0.54**	−0.06	0.70
46. Experiments involving advanced technologies are carried out on the general public without their knowledge or consent	0.08	0.07	0.11	**0.53**	0.05	0.74
3. Groups of scientists deliberately attempt to create panic about future risks because it is in their interests to do so	0.07	−0.03	−0.17	**0.49**	**0.35**	0.61
62. Advanced technology is secretly used to placate the people and suppress dissent	0.06	0.05	0.22	**0.49**	0.06	0.78
39. The government deliberately permits certain terrorist activities to occur to keep the public in a state of fear	**0.41**	−0.03	0.14	**0.47**	−0.06	0.82
54. Progress toward a cure for cancer, AIDS, and other diseases is deliberately being hindered	−0.13	0.08	0.11	**0.45**	**0.33**	0.72
13. Technology with mind-control capacities is tested on people without their knowledge or consent	0.09	0.16	0.15	**0.44**	0.02	0.75
51. Some viruses and/or diseases which many people are infected with were created in laboratories as bio-weapons	0.11	0.09	0.05	**0.43**	0.14	0.70
60. Family planning policies are part of a plot to control and limit certain populations	0.05	0.02	0.14	0.28	0.23	0.62
66. Drugs are deliberately supplied to certain communities in order to marginalize or destroy them	0.26	0.00	0.24	0.26	0.06	0.71
28. New and advanced technology which would harm current industry is being suppressed	0.20	0.10	0.01	−0.11	**0.62**	0.64
52. New and better technology is suppressed by those whose current business would be disrupted by it	0.23	0.06	0.10	−0.19	**0.62**	0.66
65. Groups of scientists ensure that only evidence which supports a pre-determined conclusion is made known to the public	−0.14	0.05	0.10	0.16	**0.57**	0.63
72. Technology is being concealed which is far in advance of what is known to the general public	0.05	0.13	−0.03	0.14	**0.57**	0.70
21. Government funded scientists manipulate evidence in order to support existing government policy	0.15	−0.01	−0.02	0.14	**0.53**	0.67
74. The media ensures that only certain information is made known to the public	0.18	−0.08	0.07	0.09	**0.45**	0.60
71. Certain groups of scientists fabricate data in support of a particular scientific theory out of self-interest	0.17	−0.09	−0.04	0.15	**0.43**	0.53
17. The government has a large amount of confidential data on every citizen without their knowledge or permission	0.08	0.00	0.18	−0.05	**0.38**	0.49
Unrotated eigenvalues	27.5	3.75	1.71	1.38	1.59	
% Of variance accounted for following rotation	19.7	15.3	21.6	22.8	18.5	

**N* = 489. Rotated loadings above 0.32 are shown in bold. GM, government malfeasance, ET, extraterrestrial cover-up, MG, malevolent global conspiracy, PW, personal wellbeing, CI, control of information, FUF, first unrotated factor*.

### Results

Exploratory factor analysis using principal-axis factoring was conducted to examine the internal structure of the 75-item measure of conspiracist beliefs. Based on the observed Eigenvalues and visual inspection of the scree plot, a six-factor solution was initially extracted. All negatively worded items were found to load onto a single factor. As there was no clear conceptual grouping other than their negative phrasing this was deemed indicative of differential item functioning rather than a true latent dimension (see Greenberger et al., 2003). Accordingly, all negatively worded items were dropped. EFA was repeated on the remaining pool of 59 items. The significance of Bartlett’s test of sphericity, χ^2^ (1711) = 23820.85, *p* < 0.001, and the size of the Kaiser–Meyer–Olkin measure of sampling adequacy, KMO = 0.97, showed that the 59 items had adequate common variance for factor analysis (Tabachnick and Fidell, 2005). Examination of initial Eigenvalues and visual inspection of the scree plot prompted investigation of the first unrotated factor as well as a five-factor solution. Parallel analysis (O’Connor, 2000) suggested a potential six-factor solution; however, given the relatively more meaningful pattern of item loadings, the more parsimonious five-factor solution was retained.

The first unrotated factor accounted for 46.64% of the total variance [Eigenvalue (λ) = 27.52], over seven times more than the second unrotated factor (λ = 3.75, 6.36% of variance accounted for). All items loaded positively with loadings ranging from 0.43 to 0.82 (see Table 1). Cronbach’s α for this factor was 0.98. Despite the strong first unrotated factor the forced five-factor solution was analyzed.

The five-factor solution explained 60.9% of the total variance. Promax oblique rotation was used based on the assumption that the factors should be related to one another. Following rotation the five factors accounted for approximately equal amounts of variance. Each factor had a minimum of five items loading substantially (0.50 or greater) and univocally on to that factor. Three items which failed to achieve a loading of 0.32 or greater on any factor were dropped. The few items that cross-loaded onto more than one factor were assigned to the factor that reflected their highest loading (factor pattern matrix loadings, item loadings for the first unrotated factor, Eigenvalues, and variance accounted for by each factor are shown in Table 1).

The pattern of loadings reflected conceptually meaningful, cohesive, and distinct groupings. Factor one, termed government malfeasance (GM), reflected allegations of routine criminal conspiracy within governments. This factor contained 16 items (α = 0.93). A second factor, termed extraterrestrial cover-up (ET), contained seven items (α = 0.94) concerning the deception of the public about the existence of aliens. A third factor, termed malevolent global (MG) conspiracies, consisted of nine items (α = 0.94) concerning allegations that small, secret groups exert total control over global events. A fourth factor, termed personal well-being (PW), consisted of 16 items (α = 0.95) relating to conspiracist concerns over personal health and liberty such as the spread of diseases and the use of mind-control technology. The fifth factor, termed control of information (CI), contained eight items (α = 0.87) relating to unethical control and suppression of information by organizations including the government, the media, scientists, and corporations.

Factor scores were computed by averaging participants’ responses to each item associated with that factor. All five factors were modestly to strongly positively intercorrelated, and all strongly correlated with the overall scale score (see Table 2).

**Table 2:** **Correlations between factor scores and overall Conspiracist Beliefs scale (long-form) score**.

Factor	ET	MG	PW	CI	Overall score
GM	0.49	0.73	0.77	0.77	0.89
ET		0.66	0.73	0.54	0.73
MG			0.84	0.74	0.90
PW				0.77	0.95
CI					0.87

**N* = 489. All correlations significant at the level *p* < 0.001 (2-tailed). GM, government malfeasance, ET, extraterrestrial cover-up, MG, malevolent global conspiracies, PW, personal wellbeing, CI, control of information*.

### Discussion

The results of exploratory factor analysis conducted on a large set of generic conspiracist scale items suggests that five important facets of conspiracism must be represented by a successful measure of conspiracist ideation: GM conspiracies; MG conspiracies; extraterrestrial conspiracies; PW-related conspiracies; CI conspiracies. Unsurprisingly, given previous evidence for the unidimensional structure of conspiracy beliefs (e.g., Goertzel, 1994; Wood et al., 2012), as well as the strong first unrotated factor and factor intercorrelations observed here, real-world conspiracy theories frequently merge these different themes. For instance, a conspiracy theory alleging that the U.S. government is secretly colluding with intelligent extraterrestrials to control and harm the human population using advanced technologies merges several of the facets identified here into a single specific theory. The factors identified here are thus not intended to reflect discrete categories of conspiracy theory but rather fundamental underlying assumptions about the world which promote beliefs in many specific conspiracy theories.

## Study 2: Factor Structure, Reliability, and Criterion-Related Validity of the 15-Item GCB Scale

### Method

#### Participants and procedure

A sample of 235 university undergraduate students was recruited from amongst several U.K. universities. Participants took part in exchange for entry into a £50 prize draw. Data were collected online via a web-based interface, and participants were asked to complete the questionnaire in a single sitting. Participants were primarily female (77.9%) and British or Irish (75.7%). Age ranged from 18 to 59 years (*M* = 24.97; SD = 8.76).

Test-retest data for the GCB scale were solicited from the first 70 participants who completed the questionnaire at Time 1, of which 42 responded and provided complete Time 2 data. Participation in the follow up study at Time 2 was voluntary; no reward was offered. The interval between Time 1 and Time 2 assessments was approximately 5 weeks. The GCB was the only measure administered at Time 2.

#### Measures

##### Generic conspiracist beliefs scale

A 15-item scale was devised (full item wording and instructions to participants can be found in Table A1 in Appendix). Three items were generated to represent each factor identified in Study 1. These five factors were intended to guide construction of the measure, ensuring content validity; however, given the observed factor intercorrelations and frequent merging of themes observed in real-world conspiracy theories, in practical usage the final GCB is primarily intended to assess conspiracist ideation as a unidimensional construct. In the interest of producing the most concise measure possible, it was not deemed necessary to include more than three items per factor. As the long-form measure designed for Study 1 was intended to reveal the important generic assumptions upon which a subsequent measure should be based rather than to provide definitive scale items, the wording of some high-loading items was modified slightly to refer more clearly to the intended GCB, some novel items were generated by merging a number of similar high-loading items into one, and some high-loading items from Study 1 were included unchanged. Participants again rated items on a 5-point Likert-type scale, with a qualitative label associated with each point (1: *definitely not true*; 2: *probably not true*; 3: *not sure/cannot decide*; 4: *probably true*; 5: *definitely true*).

##### Belief in conspiracy theories inventory

The BCTI assesses belief in 14 conspiracy theories regarding specific real-world events or organizations (α = 0.92; example item *“Government agencies in the UK are involved in the distribution of illegal drugs to ethnic minorities”*) (BCTI: Swami et al., 2010). A mean BCTI score was computed for each participant, excluding those with more than one missing data point (total valid *n* = 213).

##### Belief in 9/11 conspiracy theories

Belief in 9/11 conspiracy theories (Swami et al., 2010) was measured using a scale consisting of 17 items, each of which presents a specific conspiracy theory relating to the 9/11 terrorist attacks (α = 0.97; example item, *“Individuals within the US government knew of the impending attacks and purposely failed to act on that knowledge”*). The responses of participants with more than one missing data point were excluded (total valid *n* = 217).

##### Belief in 7/7 conspiracy theories

Belief in 7/7 conspiracy theories (Swami et al., 2011) was measured using a scale consisting of 12 items covering a range of specific conspiracy theories relating to the London bombings of July 7, 2005 (α = 0.95; example item, *“The fact that the UK government is withholding information about the 7/7 bombings is evidence of a cover-up”*). The responses of participants with more than one missing data point were excluded (total valid *n* = 220).

##### Belief in fictitious Red Bull conspiracy theories

Belief in fictitious Red Bull conspiracy theories (Swami et al., 2011) was measured using a scale consisting of 12 items covering a range of novel conspiracy theories regarding the energy drink Red Bull (α = 0.90; e.g., *“Red Bull contains illegal substances that raise the desire for the product”*). Each of these statements was made up for the purposes of previous research. The responses of participants with more than one missing data point were excluded (total valid *n* = 218).

##### Demographics

Participants were asked to indicate their age, gender, and nationality.

### Results

#### Descriptive data

A mean GCB score was computed for each participant. On the whole, participants demonstrated modest conspiracist ideation; participants were mildly skeptical but neither strongly agreed nor strongly disagreed that the conspiratorial behavior described by the GCB occurs routinely, evident from a grand mean score (*M* = 2.61; SD = 0.87) approaching the mid-point of the range of possible values (3.00). Means for each item also reflected this trend, with item means ranging from 1.88 (SD = 1.13) for the item “*Secret organizations communicate with extraterrestrials, but keep this fact from the public*” to 3.86 (SD = 1.21) for the item “*A lot of important information is deliberately concealed from the public out of self-interest*.” No floor or ceiling effects were evident; ratings for each item covered the entire range of possible scores and showed acceptable levels of skew. Overall GCB mean scores showed slight positive skew (0.24).

#### Internal reliability and factor structure

Reliability and factor analyses were restricted to participants with complete item level GCB data (*n* = 225). The overall internal reliability of the GCB scale was extremely high (α = 0.93). A maximum likelihood confirmatory factor analysis using Amos 20.0 was conducted to test the intended five-factor structure of the GCB scale. Given the scale intercorrelations observed in Study 1, a model was specified in which each factor was allowed to correlate with each other factor. Table 3 shows factor loadings for each item, and Table 4 shows factor intercorrelations. Model fit was evaluated using the following goodness-of-fit indices: the chi-square per degree of freedom (χ^2^/*df*) ratio, the comparative fit index (CFI), the goodness-of-fit index (GFI), the root mean square error of approximation (RMSEA), and the standardized root mean square residual (RMR). A χ^2^/*df* ratio of less than three indicates acceptable fit (Byrne, 2001). Concerning CFI and GFI indices, models with a value of 0.90 or above can be considered as adequately fitting. A RMSEA value below 0.08 indicates adequate fit. A RMR value of 0.05 or lower indicates close model fit. These rules of thumb are considered overly strict in some circumstances, including small sample size (*N* < 250); in such cases values approximating the above can be considered satisfactory (Marsh et al., 2004). The results indicated adequate fit. A χ^2^ value of 140.76 was obtained (*df* = 80, *p* < 0.001, χ^2^/*df* = 1.76). Other fit indices were: CFI = 0.97; GFI = 0.92; RMSEA = 0.06; RMR = 0.06. This five-factor model was a better fit for the data than a unidimensional model, according to the χ^2^ difference test for nested models (χ^2^ = 203.08, *df* = 90; χ^2^_(diff)_ = 62.32, *df*_(diff)_ = 10, *p* < 0.001).

**Table 3:** **GCB scale items and standardized factor loadings obtained with confirmatory factor analysis**.

Scale item		Factor				
		GM	MG	ET	PW	CI
1.	The government is involved in the murder of innocent citizens and/or well-known public figures, and keeps this a secret	0.75				
6.	The government permits or perpetrates acts of terrorism on its own soil, disguising its involvement	0.86				
11.	The government uses people as patsies to hide its involvement in criminal activity	0.82				
2.	The power held by heads of state is second to that of small unknown groups who really control world politics		0.77			
7.	A small, secret group of people is responsible for making all major world decisions, such as going to war		0.83			
12.	Certain significant events have been the result of the activity of a small group who secretly manipulate world events		0.91			
3.	Secret organizations communicate with extraterrestrials, but keep this fact from the public			0.75		
8.	Evidence of alien contact is being concealed from the public			0.87		
13.	Some UFO sightings and rumors are planned or staged in order to distract the public from real alien contact			0.80		
4.	The spread of certain viruses and/or diseases is the result of the deliberate, concealed efforts of some organization				0.74	
9.	Technology with mind-control capacities is used on people without their knowledge				0.69	
14.	Experiments involving new drugs or technologies are routinely carried out on the public without their knowledge or consent				0.77	
5.	Groups of scientists manipulate, fabricate, or suppress evidence in order to deceive the public					0.69
10.	New and advanced technology which would harm current industry is being suppressed					0.75
15.	A lot of important information is deliberately concealed from the public out of self-interest					0.70

**N* = 225. GM, government malfeasance, ET, extraterrestrial cover-up, MG, malevolent global conspiracies, PW, personal wellbeing, CI, control of information*.

**Table 4:** **Correlations between latent variables**.

Factor	ET	MG	PW	CI
GM	0.56	0.81	0.85	0.74
ET		0.66	0.72	0.46
MG			0.87	0.61
PW				0.79

**N* = 225. All correlations significant at the level *p* < 0.001 (2-tailed). GM, government malfeasance, ET, extraterrestrial cover-up, MG, malevolent global conspiracies, PW, personal wellbeing, CI, control of information*.

#### Short-term retest reliability

Within the test-retest sample (*N* = 42), mean GCB scores at Time 1 and Time 2 were 2.34 (SD = 0.82) and 2.28 (SD = 0.78), respectively. A paired samples *t*-test revealed that overall mean GCB scores did not change significantly over the 5-week interval; *t*(40) = 1.14, *p* = 0.26. Additionally, the correlation between mean GCB scores at Time 1 and Time 2 was positive and strong (*r* = 0.89, *p* < 0.001), indicating good test-retest reliability.

#### Criterion-related validity

The correlation between GCB and BCTI scores was strong (*n* = 202, *r* = 0.82, *p* < 0.001). GCB scores correlated strongly with scores on the measure of belief specifically in 9/11 (*n* = 206, *r* = 0.75, *p* < 0.001) conspiracy theories. In addition, mean GCB scores correlated moderately with endorsement of 7/7 (*n* = 205, *r* = 0.67, *p* < 0.001) theories, and with fictitious Red Bull theories (*n* = 209, *r* = 0.61, *p* < 0.001).

To examine the criterion-related validity of the five individual GCB facets, a multiple regression analysis was performed with belief in 9/11 conspiracy theories as the criterion variable and the scores on the five GCB factors as predictors. The overall regression model was significant; *F* = 38.06, *p* < 0.001, Adj. *R^2^* = 0.47. Of the five factors, GM, reflecting the belief that governments routinely act to harm their own citizens, was the strongest predictor (see Table 5 for standardized β values, *t*-values, and *p*-values).

**Table 5:** **Results of multiple regression analysis with GCB factors scores predicting 9/11 conspiracist beliefs**.

Factor	β	*t*	*p*
Government malfeasance	0.317	4.08	<0.001
Extraterrestrial cover-up	0.197	2.99	<0.01
Malevolent global	0.191	2.33	<0.05
Personal wellbeing	0.125	1.46	0.16
Control of information	−0.032	−0.49	0.63

### Discussion

The approximately normal distribution of mean GCB scores, centered close to the mid-point of the scale, and the absence of floor/ceiling effects or strong skew suggests that the GCB has acceptable psychometric properties and successfully captures variation in conspiracist ideation within the undergraduate student population. The results of the confirmatory factor analysis indicate that the intended five-factor structure has been retained in the 15-item GCB. Thus, the scale possesses content validity, adequately reflecting the five major components of conspiracist ideation revealed by Study 1. The strong correlation between participants’ GCB scores after a 5-week interval indicates excellent short-term test-retest reliability.

The pattern of relationships between the GCB and measures of conspiracist ideation assessing belief in certain specific event-based conspiracy theories indicates criterion-related validity; that is, mean GCB scores successfully correlate with scores on other measures of conspiracist ideation assessed concurrently. Most of these relationships were strong. The smallest relationship – with a measure assessing endorsement of an entirely novel conspiracy theory – was still substantial and in the expected direction. In addition, the individual factor scores differentially predicted endorsement specifically of 9/11 conspiracy theories, with the GM factor predicting these beliefs most strongly. This indicates that, to the extent that certain specific event-based conspiracy theories pertain more or less to particular GCB facets, the factor scores may be useful in predicting beliefs in different theories. In total these findings indicate that the GCB has acceptable criterion-related validity.

## Study 3: Convergent Validity and Additional Evidence of Criterion-Related Validity of the GCB

### Method

#### Participants and procedure

To recruit a diverse non-student sample, volunteers were requested via a public “Psychology of the Paranormal” emailing list. A total of 208 participants (91 females, 116 males, 1 participant did not provide gender information) completed the survey. Age ranged from 18 to 63 years (*M* = 40.5; SD = 13.5). The majority of participants indicated that they were British or Irish (69.2%) or U.S. (10.1%) citizens. Other nationalities made up 19.2% of the sample (1.4% did not provide nationality information). Self-selected respondents completed the questionnaire online via a web-based interface.

#### Measures

##### Generic conspiracist beliefs scale

The 15-item GCB scale developed for Study 2 was used unchanged. The internal reliability of the GCB scale in the current study was extremely high (α = 0.95).

##### Belief in conspiracy theories inventory

The BCTI was administered once again to ensure criterion-related validity in a non-student sample (BCTI: Swami et al., 2010). As in previous studies, the BCTI demonstrated high internal reliability (α = 0.93). A mean BCTI score was computed for each participant.

##### Interpersonal trust

Interpersonal trust was measured using a three-item scale (α = 0.70; example item: “*You can trust your family*”) which has been used in previous research on conspiracist belief (Goertzel, 1994). A moderate negative correlation with GCB scores was expected based on previous findings (Goertzel, 1994).

##### Anomie

Anomie (the belief that the world is generally getting worse) was measured using a three-item scale (α = 0.59; example item: “*It’s not really fair to think about having a child in today’s world*”) which has been used in previous research on conspiracist belief (Goertzel, 1994). A moderate positive correlation with GCB scores was expected based on previous findings (Goertzel, 1994).

##### Australian sheep-goat scale

Belief in the paranormal was measured using the 18-item ASGS (α = 0.96; example item, “*I believe in the existence of ESP*”) (ASGS: Thalbourne and Delin, 1993). This measure assesses belief in extrasensory perception, psychokinesis, and life after death. Participants respond to each item on a three-point scale, labeled “*True*” “*?*” (i.e., do not know), and “*False*,” resulting in a score of 2, 1, or 0 points, respectively. Higher scores reflect stronger belief in the paranormal. A moderate correlation with conspiracy theory beliefs was expected based on previous findings (Darwin et al., 2011; Swami et al., 2011).

##### Peters delusions inventory

The PDI-21 is a psychometrically validated measure of delusional ideation within the general population (PDI-21: Peters et al., 2004). Internal reliability in the current study was high (α = 0.94). The PDI assesses magical ideation, paranoia and schizotypal traits, which have previously been linked with belief in conspiracies (Darwin et al., 2011; Swami et al., 2013). In addition, one item explicitly refers to conspiracy (“*Do you ever feel there is a conspiracy against you?*”). Thus a moderate correlation with GCB scores was expected.

##### Demographics

Participants were asked to indicate their age, gender, and nationality.

### Results

#### Descriptive data

Participants generally demonstrated modest conspiracist ideation, with the grand mean (*M* = 2.22; SD = 0.92) approaching the mid-point of the range of possible values (3.00). The mean rating for each item also reflected this trend, with average item ratings ranging from 1.61 (SD = 0.94) for the item “*Secret organizations communicate with extraterrestrials, but keep this fact from the public*” to 3.52 (SD = 1.27) for the item “*A lot of important information is deliberately concealed from the public out of self-interest*.” No floor or ceiling effects were evident; ratings for each item covered the entire range of possible scores and showed acceptable levels of skew. Overall GCB scores showed mild but acceptable positive skew (0.76).

#### Criterion-related and convergent validity

The correlation between GCB and BCTI scores was positive and strong (*r* = 0.86, *p* < 0.001). Higher GCB scores correlated modestly with delusional ideation, higher anomie, lower interpersonal trust and stronger belief in the paranormal. A similar pattern of correlations emerged between BCTI scores and the personality measures (see Table 6 for all inter-scale correlations, means, and standard deviations).

**Table 6:** **Means, standard deviations, and inter-scale correlations for Study 3**.

	GCB (*M* = 2.22; SD = 0.91)	BCTI (*M* = 2.58; SD = 1.50)
Trust (*M* = 5.15; SD = 1.11)	−0.34	−0.26
Anomie (*M* = 5.06; SD = 1.29)	0.42	0.38
ASGS (*M* = 8.77; SD = 1.50)	0.67	0.67
PDI (*M* = 31.37; SD = 31.83)	0.48	0.45

*All correlations significant at the level *p* < 0.001*.

### Discussion

The descriptive statistics indicate that the GCB retains acceptable psychometric properties when used on samples not composed entirely of undergraduate students. Together with the results of Study 2, this indicates that the GCB is an appropriate measurement device both for student and non-student samples. On the whole, participants in the current study (self-selected from the general population) indicated similar, though slightly lower, levels of conspiracist ideation than the student sample reported in Study 2. The strong correlation between the GCB and the BCTI indicates that the GCB possesses criterion-related validity when used with a non-student sample; that is, higher GCB scores successfully correlate with stronger endorsement of specific conspiracy theories. The observed pattern of correlations between the GCB and measures of interpersonal trust, anomie, paranormal belief, and delusional ideation is consistent with previous findings (Goertzel, 1994; Darwin et al., 2011; Swami et al., 2011, 2013), and indicates that the GCB converges with related measures. In addition the GCB demonstrated a highly similar pattern of correlations with trust, anomie, delusions, and paranormal belief as did the BCTI, as would be expected of a successful measure of conspiracist ideation. In total these findings indicate that the GCB possesses criterion-related and convergent validity.

## Study 4: Discriminant Validity of the GCB

### Method

#### Participants and procedure

To recruit a diverse non-student sample, volunteers were requested via online forums and mailing lists. A sample of 194 participants (88 females, 105 males, 1 participant did not provide gender information) completed the survey. Age ranged from 18 to 81 years (*M* = 35.0; SD = 14.8). The majority of participants indicated that they were British or Irish (67%) or U.S. (12.9%) citizens. Other nationalities made up 18% of the sample (2.1% did not provide nationality information). Self-selected respondents completed the questionnaire via a web-based interface.

#### Measures

##### Generic conspiracist beliefs

The 15-item GCB was administered. Internal reliability was again extremely high (α = 0.95).

##### Big five inventory

The IPIP Big Five is a 50-item measure of the Big-5 factor markers (IPIP Big-Five: Goldberg, 1999). Internal reliability for each of the five factors was acceptable in the current study: openness (α = 0.75); conscientiousness (α = 0.83); extraversion (α = 0.90); agreeableness (α = 0.82); neuroticism (α = 0.85). Some previous studies have reported small but significant relationships between belief in conspiracies and higher openness and lower agreeableness, but have found no relationships with the other Big-5 traits (Swami et al., 2010, 2011, 2013). Thus we anticipate that GCB scores will correlate weakly with openness and agreeableness, and will be unrelated to the other Big-5 traits.

##### Sensation seeking

To demonstrate that the GCB is independent of theoretically independent constructs beyond the Big-5, we first selected trait sensation seeking (SSS-V: Zuckerman, 2007). Of the Big-5 traits, sensation seeking is most strongly related to extraversion (Aluja et al., 2003), which has consistently been found to be unrelated to conspiracist ideation (e.g., Swami et al., 2010, 2011, 2013); thus, no strong relationship between GCB and SSS-V scores is expected. The SSS-V is a 40-item questionnaire (α = 0.76) assessing individual differences in sensory stimulation preferences. Each question presents participants with two statements and asks that they select the statement which best reflects their own personality, e.g., “*I like ‘wild’ uninhibited parties*,” or “*I prefer quiet parties with good conversation*.” Higher scores reflect greater sensation seeking.

##### Emotional intelligence

Emotional intelligence appears to be related to Big-5 neuroticism, but weakly and unreliably correlates with agreeableness and openness (Petrides et al., 2010; TEIQue-SF: Petrides and Furnham, 2006). Thus, no strong relationship between GCB and TEIQue-SF scores is expected. The TEIQue-SF is a 30-item measure of trait emotional intelligence (α = 0.87; example item: “*Expressing my emotions with words is not a problem for me*”). Higher scores reflect greater emotional intelligence.

### Results

#### Descriptive data

Once again, participants generally demonstrated modest conspiracist ideation, with a grand mean (*M* = 2.44; SD = 1.00) slightly below the mid-point of the range of possible values (3.00). No strong skew was evident (0.47). Means for individual items reflected a similar trend as observed in Study 2 and 4, with mean endorsement ratings ranging from 1.74 to (SD = 1.02) for the item “*Secret organizations communicate with extraterrestrials, but keep this fact from the public*” to 3.68 (SD = 1.31) for the item “*A lot of important information is deliberately concealed from the public out of self-interest*.” No floor or ceiling effects were evident; ratings for each item covered the entire range of possible scores and showed acceptable levels of skew.

#### Discriminant validity

Given the sample size of the study, correlations of *r* = 0.20 will be detected at the level *p* < 0.05 with 80% confidence. Correlations between GCB scores and each of the Big-5 traits, sensation seeking, and emotional intelligence scores are shown in Table 7. No significant correlations were found between conspiracist ideation and sensation seeking, emotional intelligence, neuroticism, extraversion, agreeableness, or openness. A small significant correlation was found between conspiracist ideation and conscientiousness.

**Table 7:** **Means, standard deviations, and inter-scale correlations for Study 4**.

	GCB (*M* = 2.44; SD = 1.00)
Openness (*M* = 4.03; SD = 0.49)	−0.01
Conscientiousness (*M* = 3.13; SD = 0.70)	−0.16*
Extraversion (*M* = 3.09; SD = 0.83)	−0.01
Agreeableness (*M* = 3.85; SD = 0.62)	0.11
Neuroticism (*M* = 3.06; SD = 0.72)	−0.07
SSS-V (*M* = 20.00; SD = 5.84)	0.03
TEIQue-SF (*M* = 4.92; SD = 0.71)	−0.14

*Correlation is significant at the level *p* < 0.05.

### Discussion

As in Study 2 and 3, the GCB demonstrated acceptable psychometric properties, adequately capturing variation in conspiracist ideation in a sample of the general population. In contrast to some previous findings (Swami et al., 2010, 2011, 2013), conspiracist ideation as measured by the GCB was not related to the Big-5 traits agreeableness and openness, while a small but significant correlation emerged between greater conspiracist ideation and lower conscientiousness. While previous research looking at correlations between Big-5 traits and conspiracist ideation has failed to find this relationship, the small correlation may be explained in terms of a failure to conscientiously attend to all the available evidence, including that which conflicts with conspiracist claims. However, among the few studies to report relationships with Big-5 traits, the relationships have been small and not consistently replicated (see Swami et al., 2010, 2013). It seems most reasonable to conclude that relationships with these traits are small and somewhat unstable. Thus, conspiracist ideation cannot be described simply in terms of the Big-5 personality dimensions. As expected, GCB scores were unrelated to the remaining Big-5 traits of extraversion and neuroticism, as well as the related traits of sensation seeking and emotional intelligence. Given the power of the study to detect correlations of *r* = 0.20 or greater, the pattern of non-significant correlations observed provides evidence for the discriminant validity of the GCB scale; the GCB is either independent of other theoretically unrelated psychological constructs or is at most only weakly associated with them.

## General Discussion

The aim of this research was to create and validate a novel measure of individual differences in conspiracist ideation. Previously the dominant approach toward measuring conspiracist beliefs has involved assessing attitudes toward a small number of arbitrarily selected event-based conspiracy theories (e.g. Goertzel, 1994; Abalakina-Paap et al., 1999; Leman and Cinnirella, 2007; Swami et al., 2010; Darwin et al., 2011; Douglas and Sutton, 2011). Numerous scales have been devised, yet there has been little attempt to validate the various measures or to adopt a single measurement device, and there are concerns over the theoretical validity and practical utility of this general approach. To overcome these problems we elected to devise a measure which assesses acceptance of the generic assumptions which support belief in specific conspiracy theories.

Study 1 identified five facets of generic conspiracism through exploratory factor analysis: belief in routine governmental malfeasance; belief in the existence of MG conspiracies; belief in the existence and cover-up of extraterrestrials; concerns over the unethical CI; and belief in conspiracies infringing on PW and liberty. The existence of these dimensions is not inconsistent with other research which has found conspiracist ideation to be unidimensional (e.g., Goertzel, 1994; Swami et al., 2010; Wood et al., 2012; Bruder et al., 2013). Indeed, in the present research, the five identified dimensions of conspiracism were highly intercorrelated. We argue that the five identified facets reflect a small set of intercorrelated conspiracist assumptions which collectively serve to promote and sustain beliefs in individual conspiracy theories, resulting in a coherent network of specific conspiracist beliefs.

Based on the findings of Study 1, the GCB, a short measure sampling from each of the five identified facets of conspiracism, was produced. Studies 2, 3, and 4 provide evidence of the reliability, content, criterion, convergent, and discriminant validity of this novel measure. The results of confirmatory factor analysis reported in Study 2 demonstrated that the intended five-factor structure is retained in the 15-item GCB, ensuring that each important facet of conspiracism is successfully reflected in the measure. Overall GCB scores correlated strongly with scores on a measure of belief in various event-based conspiracy theories (the BCTI; Swami et al., 2010), as well as measures assessing belief in three specific conspiracy theories (9/11 theories, 7/7 theories, and fictitious Red Bull theories: Swami et al., 2010, 2011). The factor intercorrelations mean that in practical use the GCB can be considered a unidimensional measure; however, Study 2 also presented evidence that the individual factors may differentially predict endorsement of certain event-based conspiracy theories, to the extent that certain theories reflect certain facets of conspiracism to a greater or lesser degree. In sum, these findings indicate criterion-related validity; that is, that the five generic beliefs assessed by the GCB successfully predict endorsement of various real-world conspiracy theories.

Study 3 provided further evidence of criterion-related validity, finding GCB scores to predict general belief in various event-based conspiracy theories using an independent sample of the general public. In addition, and largely consistent with previous findings, GCB scores correlated with other related measures including low interpersonal trust, anomie, delusional ideation, paranormal belief, as well as conscientiousness (Goertzel, 1994; Darwin et al., 2011; Swami et al., 2011). Study 4 demonstrated that GCB scores were independent of unrelated psychological constructs including sensation seeking, emotional intelligence, extraversion, and neuroticism. Together, Study 3 and 4 indicate that the GCB possesses convergent and divergent validity.

It should be noted that the self-selection recruitment methods used in the current research may have resulted in unrepresentative samples; however we do not believe this to be the case. In Study 1, 3, and 4, efforts were made to recruit general-population samples diverse in terms of age, nationality, and beliefs. Study 2 allowed these samples to be compared with a sample of university students, as is typical of most psychological research. The GCB demonstrated acceptable psychometric properties when used with university students as well as when used with a self-selected sample drawn from the general population. Consistent with previous research, on the whole our samples indicated modest skepticism toward the existence of conspiracies (e.g., Goertzel, 1994; Abalakina-Paap et al., 1999; Swami et al., 2010, 2011). In addition, the relationships between the GCB and other psychological constructs were largely consistent with previous findings (e.g., Goertzel, 1994; Swami et al., 2010, 2011, 2013; Darwin et al., 2011).

Our findings show the GCB to be psychometrically at least as useful as any existing measure of conspiracist ideation which refers to specific event-based conspiracy theories (e.g., Goertzel, 1994; Swami et al., 2010; Douglas and Sutton, 2011). Given the strength of the relationships between the GCB and the BCTI (Swami et al., 2010), which refers to specific real-world conspiracy theories, it may be the case that either measure is equally useful when looking at a range of relatively homogeneous, particularly British and American, samples. However, the GCB has important advantages over these measures. By sampling from the entire range of universal and unchanging assumptions from which all specific conspiracy theories arise we have ensured content validity. In addition, by removing the context of particular historical events, the GCB offers a measurement device potentially suitable for more diverse sample populations and which will endure over time despite changing trends in popular conspiracy theorizing. We believe these practical benefits make the GCB a preferable measurement device for researchers wishing to assess conspiracist ideation.

Given the extent to which the results of exploratory factors analysis depend on the items which are created as input, it may have been possible to identify different facets of conspiracism by beginning with a different list of generic items. In Study 1, we aimed to reflect a wide range of the most popular conspiracy theories commonly discussed in the popular and academic literature. We believe the five facets we identified faithfully reflect the spectrum of popular conspiracy theorizing in Western cultures. However, different groups or cultures may be preoccupied with different types of conspiracy theories. In light of this, other measures of generic conspiracist ideation may be preferable in some contexts. Efforts to produce even more generic conspiracist measures than the GCB are underway (Bruder et al., 2013). The GCB specifies the content and objectives of some conspiracies (e.g., the suppression of new technology) while remaining non-specific about any entities or events involved in the conspiracies. In contrast, Bruder et al.’s Conspiracy Mentality Questionnaire (CMQ) remains largely non-specific in regards to both objectives and entities or events, with the exception of referring to specifically political conspiracies. Both approaches may have advantages and limitations in certain contexts. The relatively more generic approach of the CMQ may have particular advantages when studying non-Western and non-democratic cultures. Future research may seek to compare measures of conspiracist ideation across different cultures directly.

In addition to providing a validated measure of conspiracist ideation, the present findings also enhance our theoretical understanding of belief in conspiracy theories. Wood et al. (2012) demonstrated that relatively generic beliefs are important antecedents of specific conspiracy beliefs. The current findings extend upon this, identifying five underlying conspiracist beliefs and showing that these generic assumptions about the world strongly predict endorsement of various specific conspiracy theories. This is consistent with the idea that conspiracist ideation is a monological belief system (Goertzel, 1994; Wood et al., 2012) in which beliefs in many individual conspiracy theories are supported by relatively generic convictions such as the belief that governments routinely harm and deceive their citizens. Given the intercorrelations between the five conspiracist facets observed here, it is feasible that these convictions are themselves a product of even more abstract ideas, such as an essential distrust of authority. While it may be possible to construct a measure which aims to tap into these abstract sentiments, it is our opinion that the most successful measure of conspiracist ideation will be one which achieves an appropriate balance between specificity and abstractness, as we feel we have achieved with the GCB.

In sum, we believe our findings show that the GCB is a successful and psychometrically valid measure of individual differences in conspiracist ideation. The GCB has a number of advantages over existing measures of conspiracist ideation, making it a preferable measurement device for use in future research. The psychology of conspiracist beliefs is thus far a relatively under-researched topic, but recently it appears that more psychologists are turning their attention to conspiracy theories (Leman and Cinnirella, 2007; Douglas and Sutton, 2008, 2011; Bogart et al., 2010; Swami et al., 2010, 2011, 2013; Newheiser et al., 2011; Swami, 2012; Wood et al., 2012; Jolley and Douglas, 2013). It is our hope that the GCB will provide researchers wishing to assess individual differences in conspiracist ideation with a measurement device which can be used across a wide variety of empirical contexts, resulting in a consolidated and cohesive body of research.

## Conflict of Interest Statement

The authors declare that the research was conducted in the absence of any commercial or financial relationships that could be construed as a potential conflict of interest.

## Appendix

**Table A1:** **GCB scale instructions and item wording**.

Beliefs About the World There is often debate about whether or not the public is told the whole truth about various important issues. This brief survey is designed to assess your beliefs about some of these subjects. Please indicate the degree to which you believe each statement is likely to be true on the following scale: Definitely not true; Probably not true; Not sure/cannot decide; Probably true; Definitely true	
1.	The government is involved in the murder of innocent citizens and/or well-known public figures, and keeps this a secret
2.	The power held by heads of state is second to that of small unknown groups who really control world politics
3.	Secret organizations communicate with extraterrestrials, but keep this fact from the public
4.	The spread of certain viruses and/or diseases is the result of the deliberate, concealed efforts of some organization
5.	Groups of scientists manipulate, fabricate, or suppress evidence in order to deceive the public
6.	The government permits or perpetrates acts of terrorism on its own soil, disguising its involvement
7.	A small, secret group of people is responsible for making all major world decisions, such as going to war
8.	Evidence of alien contact is being concealed from the public
9.	Technology with mind-control capacities is used on people without their knowledge
10.	New and advanced technology which would harm current industry is being suppressed
11.	The government uses people as patsies to hide its involvement in criminal activity
12.	Certain significant events have been the result of the activity of a small group who secretly manipulate world events
13.	Some UFO sightings and rumors are planned or staged in order to distract the public from real alien contact
14.	Experiments involving new drugs or technologies are routinely carried out on the public without their knowledge or consent
15.	A lot of important information is deliberately concealed from the public out of self-interest
